# MicroRNA Profiling Reveals an Abundant miR-200a-3p Promotes Skeletal Muscle Satellite Cell Development by Targeting TGF-β2 and Regulating the TGF-β2/SMAD Signaling Pathway

**DOI:** 10.3390/ijms21093274

**Published:** 2020-05-05

**Authors:** Huadong Yin, Haorong He, Xiaoxu Shen, Shuyue Tang, Jing Zhao, Xinao Cao, Shunshun Han, Can Cui, Yuqi Chen, Yuanhang Wei, Yan Wang, Diyan Li, Qing Zhu

**Affiliations:** Farm Animal Genetic Resources Exploration and Innovation Key Laboratory of Sichuan Province, Sichuan Agricultural University, Chengdu 611130, China; yinhuadong@sicau.edu.cn (H.Y.); hehaorong@stu.sicau.edu.cn (H.H.); shenxiaoxu@stu.sicau.edu.cn (X.S.); tangshuyue@stu.sicau.edu.cn (S.T.); zhaojing@stu.sicau.edu.cn (J.Z.); caoxinao@stu.sicau.edu.cn (X.C.); hanshunshun@stu.sicau.edu.cn (S.H.); cuican123@stu.sicau.edu.cn (C.C.); chenyuqi@stu.sicau.edu.cn (Y.C.); weiyuanhang@stu.sicau.edu.cn (Y.W.); wangyan519@sicau.edu.cn (Y.W.); diyanli@sicau.edu.cn (D.L.)

**Keywords:** RNA-seq, skeletal muscle satellite cells, miR-200a-3p, TGF-β2/SMAD pathway, differentiation, proliferation, apoptosis

## Abstract

MicroRNAs (miRNAs) are evolutionarily conserved, small noncoding RNAs that play critical post-transcriptional regulatory roles in skeletal muscle development. Chicken is an optimal model to study skeletal muscle formation because its developmental anatomy is similar to that of mammals. In this study, we identified potential miRNAs in the breast muscle of broilers and layers at embryonic day 10 (E10), E13, E16, and E19. We detected 1836 miRNAs, 233 of which were differentially expressed between broilers and layers. In particular, miRNA-200a-3p was significantly more highly expressed in broilers than layers at three time points. In vitro experiments showed that miR-200a-3p accelerated differentiation and proliferation of chicken skeletal muscle satellite cells (SMSCs) and inhibited SMSCs apoptosis. The transforming growth factor 2 (*TGF-β2*) was identified as a target gene of miR-200a-3p, and which turned out to inhibit differentiation and proliferation, and promote apoptosis of SMSCs. Exogenous TGF-β2 increased the abundances of phosphorylated SMAD2 and SMAD3 proteins, and a miR-200a-3p mimic weakened this effect. The TGF-β2 inhibitor treatment reduced the promotional and inhibitory effects of miR-200a-3p on SMSC differentiation and apoptosis, respectively. Our results indicate that miRNAs are abundantly expressed during embryonic skeletal muscle development, and that miR-200a-3p promotes SMSC development by targeting TGF-β2 and regulating the TGF-β2/SMAD signaling pathway.

## 1. Introduction

Muscle is an important component of animal bodies, including human bodies. Skeletal muscle accounts for about 40–60% of the body weight of adult animals. Muscle fibers are the functional units of muscle and their numbers are mostly fixed in the embryonic stage, and postnatal muscle growth is mainly due to hypertrophy and hyperplasia of the muscle fibers, which is accompanied by the proliferation and differentiation of activated skeletal muscle satellite cells (SMSCs) [1]. In chicken, the primary muscle fibers were shown to appear on day 6 of the incubation period, the secondary muscle fibers formed mainly on days 12–16 of embryo development, and differentiation of the muscle fibers was completed on day 18 of incubation [2]. During embryonic myogenesis, muscle fibers are derived from mesoderm-derived structures under a tightly-regulated multistep process that is controlled by regulatory factors [3]. 

Noncoding RNAs have been demonstrated to play vital roles in post-transcriptional regulation of myogenesis and in the development of skeletal muscle [4]. MicroRNAs (miRNAs) are small approximately 22-nucleotide long highly-conserved endogenous noncoding RNAs that bind to the 3′-untranslated regions (UTRs) of target mRNAs and negatively regulate gene expression [5]. MiRNAs function as master regulators in fundamental cellular processes, including proliferation, apoptosis, and differentiation [6]. Several miRNAs that play crucial roles in myogenesis have been recorded. Among them, members of the miR-1/miR-206 and miR-133a/miR-133b families are well-studied myogenic miRNAs that regulate skeletal muscle myogenesis, including myoblast/satellite cell proliferation and differentiation [7].

Xu et al. [8] identified 25 miRNAs in chicken embryo and adult tissues, and miR-757 was deemed to be chicken specific. Li et al. [9] evaluated miRNA function in chicken skeletal muscle development by the miRNA microarray analysis and detected 33 novels and 189 known chicken miRNAs. The development of high-throughput sequencing technologies and bioinformatics has rapidly increased the numbers of reported chicken miRNAs. For example, Ouyang et al. [10] identified 921 miRNAs (733 known and 188 novels) in breast muscle tissues of two-tail samples from Recessive White Rock (higher body weight) and Xinghua (lower body weight) chickens at seven weeks. Subsequently, Khatri et al. [11] identified nine differentially expressed miRNAs in breast muscle in pedigree male broilers (rapid growth) compared with Barred Plymouth Rock chickens (slow growth); eight were upregulated and one, miR-206, was downregulated in the pedigree male broilers.

It is worth noting that in the past few decades, high intensity artificial breeding has led to major differences in the growth and development between layers and broilers. Even under the optimal growth conditions, layers are much smaller than broilers. At six weeks of age, the weight of broilers can even be five times than that of layers. Therefore, the specialized layers and broilers are the ideal model system for studying the mechanism of myogenesis [9,10,11,12]. In this study, we performed high-throughput small RNA deep sequencing on breast muscle tissues from 12 standardized broilers (Ross 308, higher body weight) and 12 standardized layers (White Longhorn, lower body weight) at embryonic day 10 (E10), E13, E16, and E19 to identify miRNAs that may play roles in embryonic myogenesis of chicken skeletal muscle. In short, the purpose of this study is to explore the miRNAs that have roles in the development of chicken skeletal muscle embryogenesis, and to study the specific functions and pathways of miRNAs that affect myogenesis.

## 2. Results

### 2.1. Overview of the MiRNA Deep Sequencing Data

We obtained 11,727,190 to 19,127,517 raw reads among the eight sets (three repetitions per set). After removing reads that were < 18 and > 30 nucleotides (nt) long, the remaining 10,888,197 to 18,153,166 reads were considered as clean reads. The ratio of clean reads occupancy raw reads from 90.45% to 97.32% and the sequencing quality (Q30) was > 85% (Table S1). Noncoding RNA sequences, including ribosomal RNA (rRNA), transfer RNA (tRNA), small nuclear RNA (snRNA), and small nucleolar RNA (snoRNA) sequences, as well as repeated sequences were removed and the remaining reads were considered as candidate miRNAs (Table S2).

### 2.2. Characterization of Identified MiRNAs

**Figure 1:**
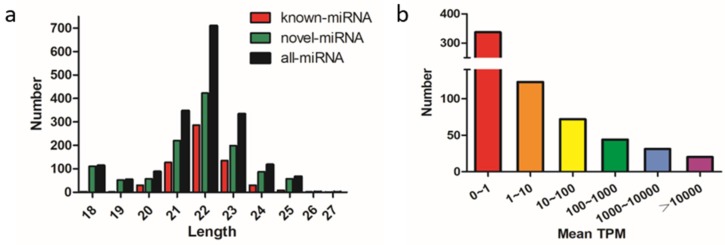
Characterization of known and predicted novel microRNAs (miRNAs) in chicken breast muscle. (**a**) Length distribution and abundance of miRNAs sequences, clean reads of 18–27 nt for all four groups were assessed, and the most abundant size class was 22 nt; (**b**) distribution of miRNAs with 0 to > 10,000 mean transcripts per million (TPM).

The remaining clean reads were mapped to the chicken reference mature miRNA collections, and a total of 1836 miRNAs were identified among the broiler and layer reads; 627 were known miRNAs and 1209 were predicted novel miRNAs. The length distribution of the known and novel miRNAs showed that most were 21–23 nt long, with the majority being 21 nt (Figure 1a). Most of the miRNAs had mean transcripts per million (TPM) values of < 1, and 20 miRNAs had mean TPM values >10,000, implying that only a few of the detected miRNAs were highly expressed and potentially involved in muscle development in chicken (Figure 1b). The top 10 most abundant known miRNAs are listed in Table 1. MiR-148a-3p was the most abundant of mean TPM values with 33,4272 and 35,7031 in the broiler and layer libraries, respectively. Except for miR-199-3p and miR-99a-5p, the mean TPM values of the other eight miRNAs were higher in broilers than these in layers.

### 2.3. Characterization of Differentially Expressed MiRNAs

**Figure 2:**
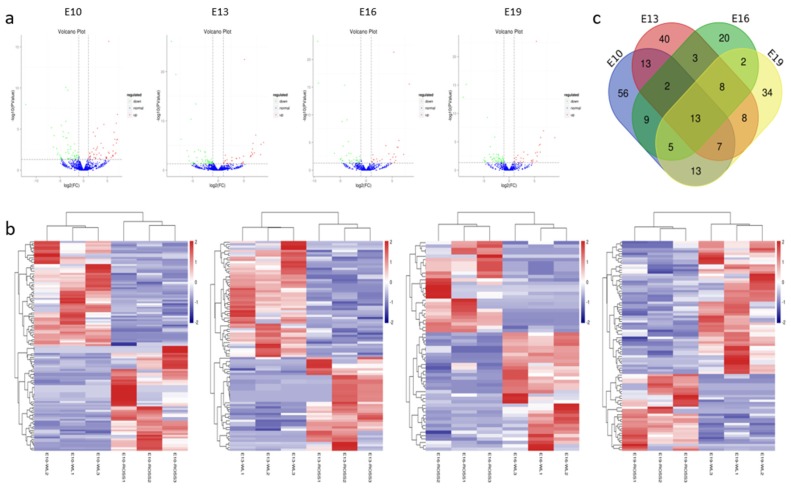
Characterization of the differentially expressed (DEMs). (**a**) The difference in expression levels of miRNAs between broilers and layers at different time points. (**b**) Heatmap of differentially expressed miRNAs in each paired group (broilers vs. layers). (**c**) Venn diagrams of DEMs in six comparison group (*n* = 233; *p* < 0.05, fold change ≥ 2).

Differentially expressed miRNAs (DEMs) were identified using two criteria, fold change (FC) ≥ 2 and *p* < 0.05, between broilers and layers at four time points (E10, E13, E16, and E19). A total of 233 DEMs were detected (Table S3 and Figure 2a). Among them, 118 (59 upregulated and 59 downregulated), 94 (42 upregulated and 52 downregulated), 62 (27 upregulated and 35 downregulated), and 90 (33 upregulated and 57 downregulated) were detected at E10, E13, E16, and E19, respectively. Clustering analysis indicated that the miRNA expression patterns were different in broilers and layers (Figure 2b). In addition, 150 of the DEMs were differentially expressed at one of the time points, whereas the other 83 were differentially expressed at multiple time points (Figure 2c).

### 2.4. Target Gene Prediction and Function Annotation

A total of 25,086 consensus target genes of all the DEMs were predicted using miRanda and RNAhybrid (Table S4); among them, 18,353 target genes were annotated (Table S5). The Gene Ontology (GO) annotation and Kyoto Encyclopedia of Genes and Genomes (KEGG) pathway analysis were performed to identify functional modules of the DEMs target genes. The GO annotation results showed that the most abundant terms included cellular processes (material transport and catabolism, cell movement, cell growth and death, and cellular communication), biological regulation, developmental processes, and growth (Figure S1a). The KEGG pathway analysis revealed that most of the targets were involved in the Wnt signaling, TGF-β signaling, Notch signaling, and MAPK signaling pathways (Figure S1b).

### 2.5. Construction of miRNA–mRNA Interaction Network

**Figure 3:**
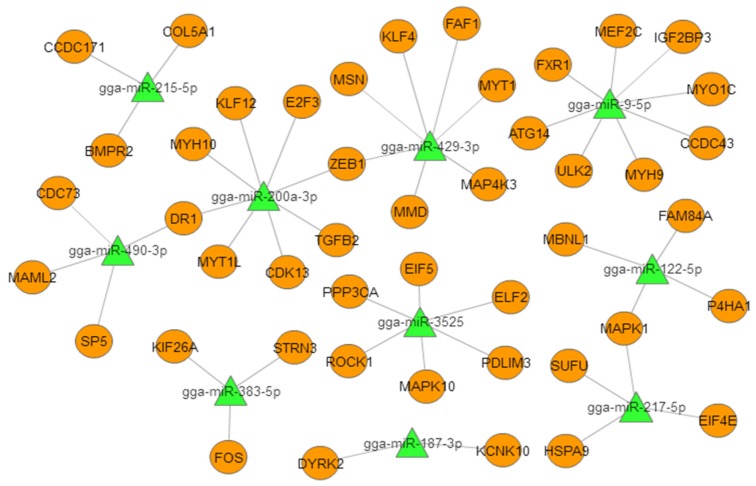
Proposed miRNA–mRNA interaction network of the 10 most abundant differentially expressed miRNAs (DEMs) with their potential target mRNAs. The miRNAs are represented by green triangles and targets are represented by orange circles.

To better understand the role of miRNAs in chicken embryonic muscle development, we selected the top 10 DEMs for biofunctionality validation (Table 2). We constructed a miRNA–mRNA (target gene) interactive network based on the predicted relationships between the top 10 DEMs and their target genes (Figure 3). The results showed these DEMs targeted important genes that are known to be involved in muscle development. In particular, miR-9-5p targeted *MEF2C* and *IGF2BP3*, miR-200a-3p targeted *TGF-β2* and *MYH10*, and miR-3532 targeted *PPP3CA* and *PDLIM3*, which suggested that these miRNAs may play critical roles in muscle growth and development.

### 2.6. Experimental Validation of Selected DEMs

**Figure 4:**
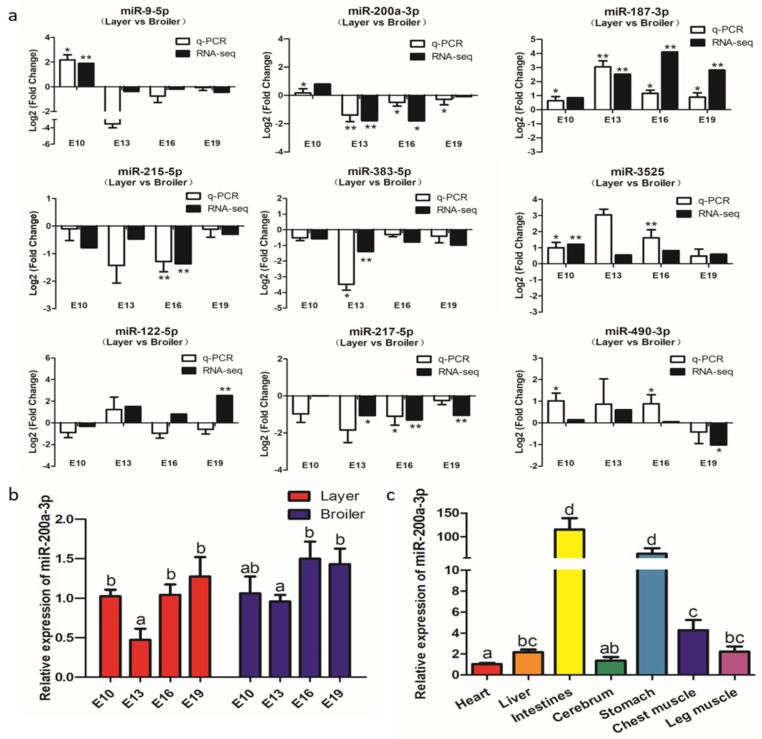
Experimental validation of differentially expressed miRNAs (DEMs) by qRT-PCR. (**a**) qRT-PCR validation of nine differentially expressed miRNAs in four comparison groups. (**b**) Expression levels of miR-200a-3p in breast muscle of broilers and layers at four embryonic ages. (**c**) Expression levels of miR-200a-3p in tissues of broiler chickens, the mRNA level in the heart as control. Data are presented as mean ± SEM (*n* = 3). *p < 0.05; **p < 0.01.

To verify the accuracy of the RNA-seq data, we designed primers and analyzed the expression of nine DEMs, namely miR-9-5p, miR-200a-3p, miR-187-3p, miR-215-5p, miR-383-5p, miR-3525, miR-122-5p, miR-122-5p, and miR-490-3p at the E10, E13, E16, and E19 by qRT-PCR. The results showed that the expression patterns of these DEMs (except miR-122-5p) were almost identical to the expression patterns determined from the RNA-seq data (Figure 4a), indicating that the RNA-seq data are reliable and suitable for further analysis.

The qRT-PCR and RNA-seq results confirmed that miR-200a-3p was significantly highly expressed in broilers compared to layers at E13, E16, and E19 (Figure 4a). MiR-200a-3p was highly expressed at E10, its expression decreased at E13, and then increased at E16 and E19 (Figure 4b). MiR-200a-3p also was expressed in seven chicken tissues (Figure 4c), and was especially highly expressed in the muscle tissues, namely smooth muscle (intestine and stomach) and skeletal muscle (breast muscle and leg muscle). These results suggested that miR-200a-3p played an important role in skeletal muscle development.

### 2.7. MiR-200a-3p Promotes Differentiation of Sketetal Muscle Satellite Cells (SMSCs)

**Figure 5:**
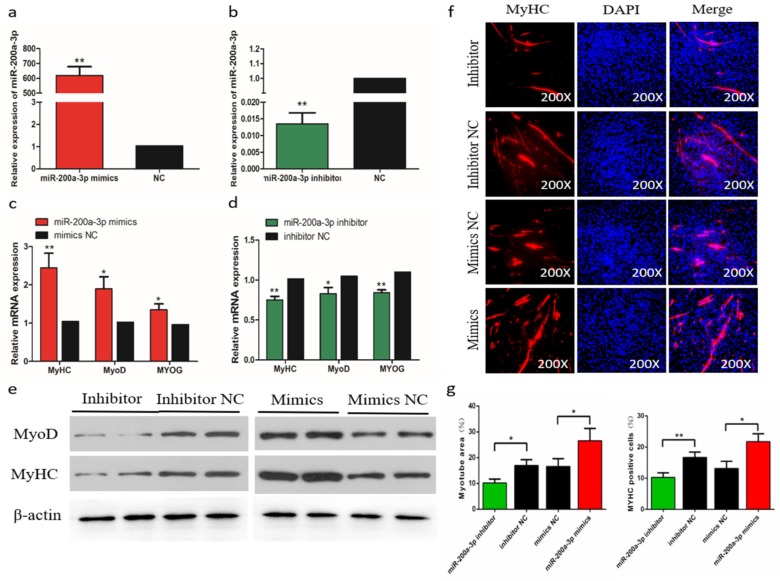
Effects of miR-200a-3p on the differentiation of skeletal muscle satellite cells (SMSCs). (**a**,**b**) Expression of miR-200a-3p in SMSCs was monitored using qRT-PCR following transfection with a miR-200a-3p inhibitor or mimic. (**c**,**d**) The relative expressions of myogenic determination 1 (MyoD1), myosin heavy chain (MyHC), and myogenin (MyoG) mRNA in SMSCs differentiated for 48 h. (**e**) Western blot detects protein levels of myogenic marker genes after inhibition and overexpression of miR-200a-3p. (**f**) Representative images of immunofluorescent staining of differentiated SMSCs (200×). Myosin: Red, a molecular marker of myogenesis; DAPI: Blue, cell nuclei; Merge: The fusion of SMSCs into primary myotubes. (**g**) Myotube area (%) and MYHC positive cells (%) after transfection of miR-200a-3p mimics, miR-200a-3p inhibitors, or negative control (NC). Data are expressed as mean ± SEM (*n* = 3). *p < 0.05; **p < 0.01 vs. NC.

The efficiency of miR-200a-3p interference and overexpression in chicken skeletal muscle satellite cells (SMSCs) was 83 and 619 times than the control, respectively (*p* < 0.01; Figure 5a,b). To determine the role of miR-200a-3p in the differentiation of chicken SMSCs, we measured the expression changes of myogenic differentiation marker genes, including myogenic determination 1 (*MyoD1*), myogenin (*MyoG*), and myosin heavy chain (*MyHC*). The results showed that the increased expression of miR-200a-3p led to increased expression of the myogenic differentiation marker genes (*p* < 0.05; Figure 5c), whereas the expression levels of these genes were significantly decreased in the miR-200a-3p knockdown SMSCs (*p* < 0.05; Figure 5d). The similar effects on abundances of the MyoD1 and MyHC protein were found in SMSCs treated with a miR-200a-3p mimic or inhibitor (*p* < 0.05; Figure 5e). The myosin immunofluorescence results showed that overexpression of miR-200a-3p promoted myotube formation, whereas the knockdown of miR-200a-3p inhibited SMSCs differentiation. In addition, the myotube area increased significantly after miR-200a-3p overexpression, and the opposite was true for SMSCs treated with a miR-200a-3p inhibitor (*p* < 0.05; Figure 5f,g). Overall, these results indicated that miR-200a-3p promoted the differentiation of chicken SMSCs.

### 2.8. MiR-200a-3p Promotes Proliferation and Inhibits Apoptosis of SMSCs

**Figure 6:**
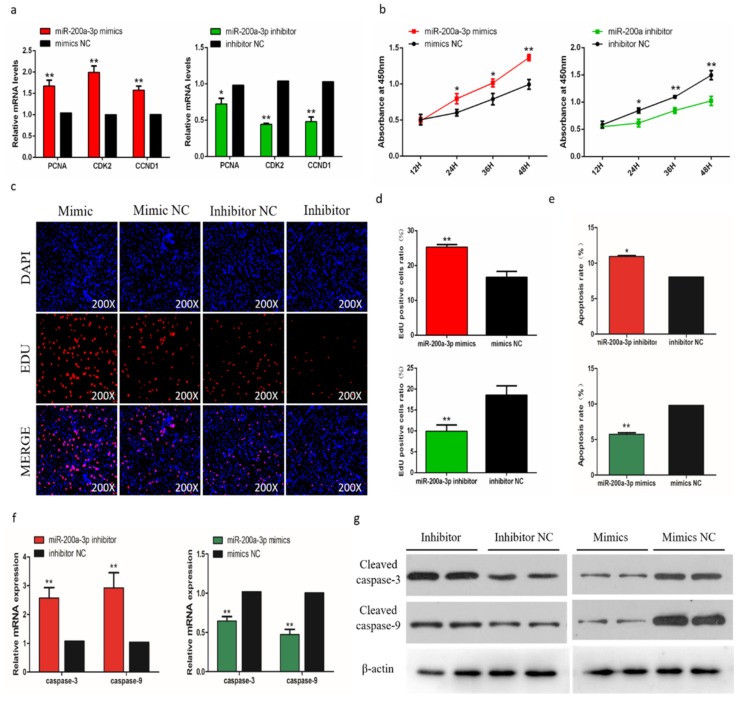
Effects of miR-200a-3p on the apoptosis of skeletal muscle satellite cells (SMSCs). (**a**) Detection of mRNA levels of SMSC cell proliferation-related genes after overexpression and inhibition of miR-200a-3p by qRT-PCR. (**b**) Cell Counting Kit-8 (CCK-8) detects SMSCs after overexpression and inhibition of miR-200a-3p. (**c**,**d**) EdU staining and chicken SMSCs proliferation rate after transfection of miR-200a-3p mimics and inhibitors in SMSCs. The results are shown as an average scanning electron microscope, and the data represent at least three independent analyses. Student’s t-test was used to compare the expression levels between groups. (**e**) Scattergram and the rate of apoptosis in SMSCs transfected with miR-200a-3p mimics or miR-200a-3p inhibitors as analyzed using flow cytometry following staining with annexin V and PI. (**f**,**g**) Abundance of mRNA and proteins of caspase-3 and caspase-9 in SMSCs transfected with miR-200a-3p inhibitors and miR-200a-3p mimics as determined by the use of qPCR and Western blot analysis. Data are expressed as mean ± SEM (*n* = 3). *p < 0.05; **p < 0.01 vs. NC.

To evaluate the biological effects of miR-200a-3p on SMSCs proliferation and apoptosis, we measured the expression of proliferation marker genes (PCNA, CDK2, and CCND1) during cell proliferation, and the results showed that genes expressions were suppressed after the miR-200a-3p inhibition. In contrast, the addition of miR-200a-3p mimics can promote these genes expressions (Figure 6a). The trend of CCK-8 test results showed that after miR-200a-3p knockdown, the proliferation of SMSCs was significantly reduced and the opposite result was induced by miR-200a-3p overexpression in cells (Figure 6b). The EdU analysis also showed that after miR-200a-3p knockdown, the proliferation of SMSC was significantly decreased and overexpression of miR-200a-3p significantly promoted the number of EDU positive cells (Figure 6c,d). In addition, we analyzed the apoptosis rate by flow cytometry and the results showed that the apoptosis rate of SMSCs was reduced in the miR-200a-3p overexpressed cells, while the inhibitory effect of miR-200a-3p was reversed (Figure 6e). We also monitored the expression of apoptosis-related caspase genes CASP3 and CASP9 in SMSCs. After miR-200a-3p overexpression, the expression of CASP3 and CASP9 mRNA and protein decreased, while increased in the miR-200a-3p knockdown cells (Figure 6f,g).

### 2.9. TGF-β2 is a Target Gene of MiR-200a-3p

**Figure 7:**
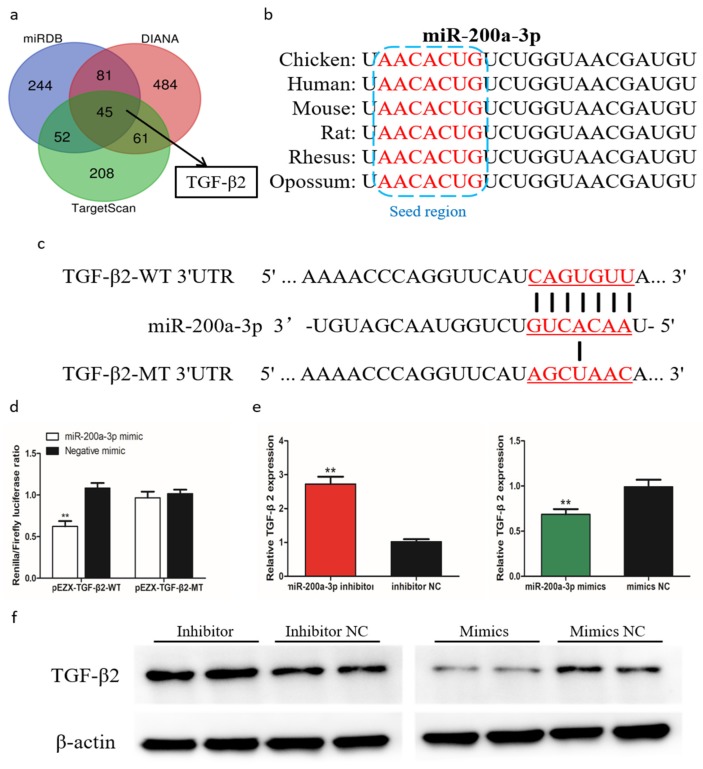
MiR-200a-3p directly targets *TGF-β2* in skeletal muscle satellite cells (SMSCs). (**a**) Prediction of target genes using TargetScan, miRDB, and Diana. (**b**) Seed region of miR-200a-3p. (**c**) Dual-luciferase reporter gene (pEZX-FR02) with wild type (pEZX-*TGF-β2*-WT) or mutant (pEZX-*TGF-β2*-MT). (**d**) Luciferase assays were performed by cotransfection of wild type or mutant TGF-β2 3′ UTR with a miR-200a-3p mimic or mimic-NC in SMSCs. (**e**,**f**) After transfection of miR-200a-3p mimics, miR-200a-3p inhibitors or NC, the expression of TGF-β2 was determined by q-PCR and Western blot. Data are expressed as mean ± SEM (*n* = 3). *p < 0.05; **p < 0.01.

To further understand the mechanism by which miR-200a-3p regulates SMSCs proliferation, differentiation, and apoptosis, we predicted the target genes of miR-200a-3p using three online software tools (TargetScan, miRDB, and Diana). Forty-five target genes were predicted by all three software tools, including *TGF-β2* (Figure 7a). The seed sequence alignment indicated that miR-200a-3p was highly conserved in various species, including human, mouse, rat, possum, and chicken (Figure 7b). To verify the relationship of miR-200a-3p and *TGF-β2* in chicken SMSCs, we performed a double luciferase reporting experiment. A double luciferase reporter (pEZX-FR02) with wild type (pEZX-*TGF-β2*-WT) or mutant (pEZX-*TGF-β2*-MT) was constructed using the *TGF-β2* 3′-UTR sequence at the 3′-end of firefly luciferase in DF-1 cells, respectively (Figure 7c). After cotransfection of the dual luciferase reporter gene and miR-200a-3p mimics, firefly luciferase activity in the pEZX-*TGF-β2*-WT plasmid was reduced, but no change was detected in cells cotransfected with the mutant reporter gene (Figure 7d). We found that *TGF-β2* mRNA expression and TGF-β2 protein abundance were reduced in SMSCs with overexpressing miR-200a-3p, whereas they were increased in miR-200a-3p inhibited SMSCs (Figure 7e,f). These results confirmed that *TGF-β2* was a target gene of miR-200a-3p.

### 2.10. TGF-β2 Negatively Regulates Differentiation of SMSCs

**Figure 8:**
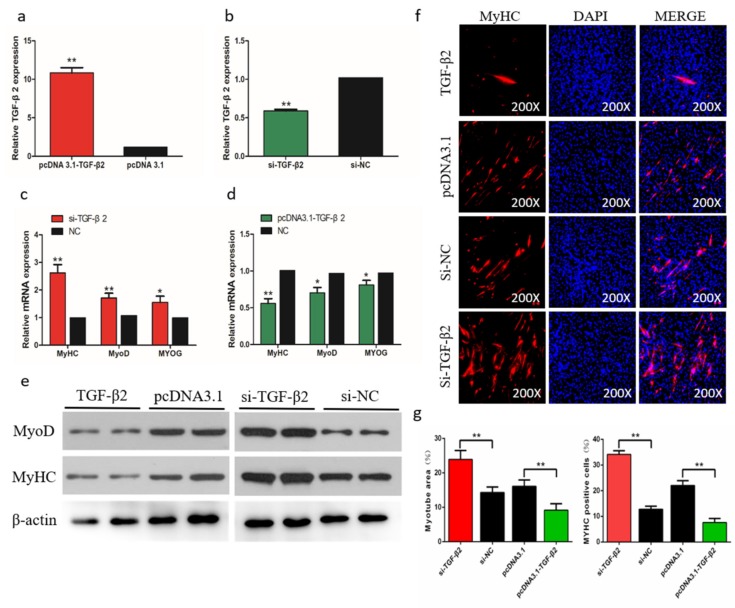
Effects of TGF-β2 on differentiation of skeletal muscle satellite cells (SMSCs). (**a**,**b**) After overexpression or interference with TGF-β2, qRT-PCR was used to detect the expression of TGF-β2 in SMSCs. (**c**,**d**) The relative expressions of MyoD, MyHC, and MyoG mRNA in SMSCs differentiated for 48 h. (**e**) Western blot detects protein levels of myogenic marker genes after inhibition and overexpression of TGF-β2. (**f**) Representative images of immunofluorescent staining of differentiated SMSCs (200×). Myosin: Red, a molecular marker of myogenesis; DAPI: Blue, cell nuclei; Merge: The fusion of SMSCs into primary myotubes. (**g**) Myotube area (%) and MYHC positive cells (%) after transfection of si-TGF-β2, pcDNA3.1-TGF-β2, or NC. Data are expressed as mean ± SEM (*n* = 3). *p < 0.05; **p < 0.01 vs. NC.

To determine the role of TGF-β2 in differentiation of chicken SMSCs, we constructed effective overexpression vectors and short interfering RNAs (siRNAs) of *TGF-β2* to treat the SMSCs (*p* < 0.01, Figure 8a,b). The expression levels of *MyoD1*, *MyoG*, and *MyHC* increased after *TGF-β2* knockdown in SMSCs but decreased in cells with overexpressing *TGF-β2* (*p* < 0.05, Figure 8c,d). The abundance of MyoD1 and MyHC followed a similar trend with the *TGF-β2* treatments (Figure 8e). The immunofluorescence results that overexpression of *TGF-β2* inhibited myoblast fusion to form myotubes, whereas interference of *TGF-β2* promoted this process in SMSCs (Figure 8f,g). Overall, these results indicated that TGF-β2 inhibited the differentiation of SMSCs.

### 2.11. TGF-β2 has a Negative Effect on the Proliferation and a Positive Effect on Apoptosis

**Figure 9:**
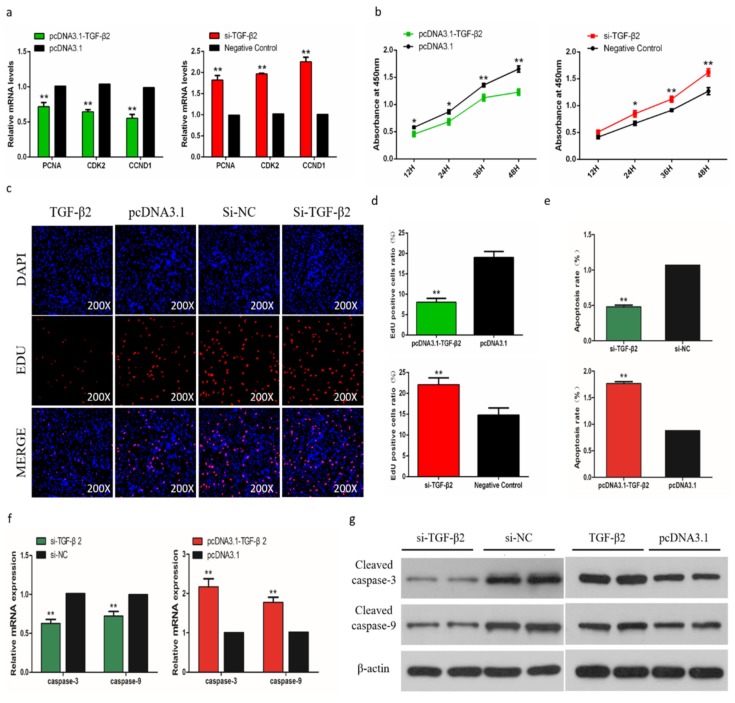
Effects of TGF-β2 on apoptosis of skeletal muscle satellite cells (SMSCs). (**a**,**b**) Scattergram and the rate of apoptosis in SMSCs transfected with promoting or inhibiting TGF-β2 expression as analyzed using flow cytometry following staining with annexin V and PI. (**c**,**d**) Abundance of mRNA and proteins of caspase-3 and caspase-9 in SMSCs transfected by the use of qPCR and Western blot analysis. Data are expressed as mean ± SEM (*n* = 3). *p < 0.05; **p < 0.01 vs. NC.

In order to assess the biological effect of TGF-β2 on the proliferation of SMSCs, we detected the expression of marker genes and the trend of CCK-8 during cell proliferation. The results showed that after knockdown of TGF-β2, the proliferation of SMSC was significantly promoted, and overexpression of TGF-β2 induced the opposite result (Figure 9a,b). The EdU analysis also showed similar results (Figure 9c,d). To determine the role of TGF-β2 in the apoptosis of SMSCs, we used a flow cytometry to analyze the apoptosis after regulating the abundance of TGF-β2 in SMSCs. The results showed that the percentage of apoptotic was significantly increased in TGF-β2 overexpressed cells, while knocking down TGF-β2 reduced the level of apoptosis in SMSCs (Figure 9e). We also detected the CASP3 and CASP9 regulators associated with apoptosis in SMSCs. Changes in mRNA expression and protein abundance of these regulators also indicated that TGF-β2 promoted the apoptosis of SMSCs (Figure 9f,g). 

### 2.12. MiR-200a-3p Regulates Differentiation and Apoptosis of SMSCs via TGF-β2/SMAD Signaling

**Figure 10:**
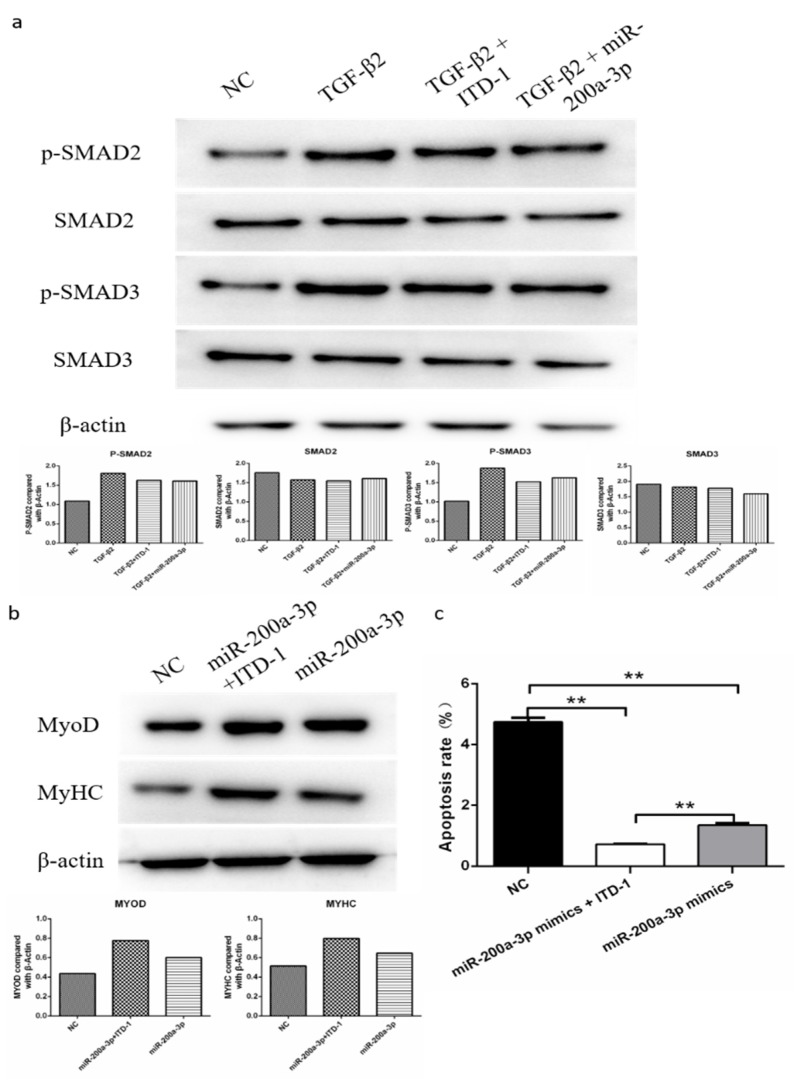
MiR-200a-3p regulates differentiation and apoptosis of skeletal muscle satellite cells (SMSCs) via the TGF-β/SMAD pathway. (**a**) After overexpression of TGF-β2/TGF-β2 + miR-200a-3p/TGF-β2 + ITD-1, the protein levels of TGF-β/SMAD pathway genes were detected using Western blotting. (**b**,**c**) Effect of miR-200a-3p mimics on differentiation and apoptosis of SMSC cells in the absence or presence of ITD-1. Data are expressed as mean ± SEM (*n* = 3). *p < 0.05; **p < 0.01 vs. NC.

To find out if TGF-β2/SMAD signaling is involved in the regulation of differentiation and apoptosis of SMSCs by miR-200a-3p, we determined the abundance of the phosphorylated SMAD2/3 protein (p-SMAD2/3) and total SAMD3 protein in SMSCs. The cells were cultured in the presence and absence of pcDNA3.1-*TGF-β2*, *TGF-β2* inhibitor, or miR-200a-3p mimic, respectively. Western blot results showed that exogenous TGF-β2 increased the levels of p-SMAD2 and p-SMAD3, whereas the SMAD2 and SMAD3 expression levels were unaltered in SMSCs. However, cotransfection of pcDNA3.1-*TGF-β2* and miR-200a-3p mimic restored the effects of p-SMAD2 and p-SMAD3 expression induced by TGF-β2 (Figure 10a). To further confirm our results, we used a specific *TGF-β2* inhibitor (inducer of TGFβ type II receptor degradation-1; ITD-1) to treat SMSCs. The results suggested that exogenous miR-200a-3p increased cell differentiation capacity and decreased cell apoptosis rate, but these effects were inhibited by a simultaneous treatment with the pharmacological inhibitor ITD-1 (Figure 10b,c). Overall, these results showed that the regulation of differentiation and apoptosis of SMSCs by miR-200a-3p was dependent on the TGF-β2/SMAD signaling pathway.

## 3. Discussion

Skeletal muscle is essential for all animals, including humans. Loss of skeletal muscle function and quantity in the human body accelerates catabolic metabolism, and influences the effect of cancer treatment, acquired immunodeficiency syndrome, metabolic syndrome and other diseases, and eventually leads to increased morbidity and mortality [13]. Skeletal muscle also is the most economically valuable tissue in meat-producing animals, and its quantity and quality directly determine production efficiency [14].

Chicken is one of the best models to study embryonic skeletal muscle development in vertebrates, because its developmental anatomy is very similar to that of mammals, as well as the in vitro development of the embryos allows easy sampling and has low cost [15]. In addition, high-strength artificial selection in the past few decades has produced highly significant differences in muscle development between layer and broiler chickens [12]. Although a number of skeletal muscle-related miRNAs have been identified in chicken, many miRNAs that regulate embryonic chicken skeletal muscle development have not yet been discovered because of limitations in sequencing technology and sampling time points. Therefore, we performed a high-throughput RNA-seq analysis to identify and annotate a considerable number of miRNAs in chicken embryonic breast muscle. To our knowledge, this is the first overview of the types and relative abundances of miRNAs at four embryonic developmental stages of skeletal muscle tissue between broilers and layers.

We identified 1836 miRNAs from all the sequencing libraries; 627 were known miRNAs and 1209 were novel miRNAs. Most of the miRNAs were 20–24 nt long, and within this range most were 22 nt, followed by 21 and 23 nt, which is consistent with the known lengths of miRNAs [16]. Generally, the more abundant a miRNA transcript is, the more likely it is to function. The most abundant miRNA detected in the breast muscle tissue was miR-148a-3p, which is known to promote skeletal muscle development in mice [17], cattle [18], and chicken [19]. Muscle-specific miRNAs such as miR-1a-3p and miR-206, which are considered to be important for myoblast differentiation and proliferation [7], also were expressed abundantly in the chicken embryonic skeletal muscle. In addition, miR-199-3p, miR-26a-5p, and miR-21-5p, which were in the top 10 most abundantly expressed miRNAs, have been reported to regulate skeletal muscle growth and development [20,21,22]. These results supported the validity of our RNA-seq data between layers and broilers to identify muscle-related miRNAs during different embryonic development stages.

We identified 233 miRNAs that were differentially expressed between layers and broilers, and 83 of them were identified at more than one development stage. Compared with the results of Li et al. [9], we found several additional miRNAs involved in muscle development processes. To understand the cellular and physiological mechanisms involving these DEMs, we predicted the target genes of the DEMs and annotated them. The results showed that the predicted target genes were associated with many biological processes such as cell movement, cell growth and death, and cellular communication, and were involved mainly in muscle growth-related signaling pathways including the Wnt, TGF-β, Notch, and MAPK pathways. Therefore, the DEMs are likely to be closely related with muscle growth and development in chicken.

Among the 83 DEMs, we found that miR-200a-3p was very highly (top 2) expressed in the chicken embryonic breast muscle. Both of the sequencing data and qRT-PCR results confirmed that miR-200a-3p were differentially expressed at E13, E16, and E19, and its expression was significantly higher in broilers at the three time points. Similarly, Li et al. [9] found that miRNA-200a was differentially expressed between broilers and layers, and its expression was higher in broilers. Hence, we postulated that miR-200a-3p may be a positive regulator for chicken skeletal muscle development. To test this hypothesis, we investigated the role of miR-200a-3p in chicken SMSCs by modulating miR-200-3p expression in vitro. The results confirmed that miR-200a-3p promoted differentiation and proliferation of SMSCs, and inhibited apoptosis of SMSCs in chicken. These results are consistent with the role of miR-200a-3p in human cardiac muscle [23] and airway smooth muscle cells [24]. In addition, it was observed that miR-200a-3p is a negative factor affecting cell proliferation in human cancer. In human hepatocellular carcinoma (HCC), the circ-ZEB1.33-miR-200a-3p-CDK6 regulatory axis can regulate HCC cell proliferation [25]. In gastric cancer (GC) tissues, miR-200a-3p plays a cancer suppressing role in GC by targeting KLF12 [26]. In renal cell carcinoma (RCC), the miR-200a-3p/CBL regulatory axis is a new mechanism under pathogenesis [27]. After using high-throughput sequencing to analyze the ovarian tissues of chickens with lower and higher egg rates, Wu et al. found that miR-200a-3p may play a special central role in the reproductive regulation [28]. Although Jiang et al. detected temporal expression profile of miR-200a in chicken breast muscle, and found that Grb2 is one of its target gene, they did not prove the function of miR-200a in chicken muscle through the experiment [29]. Therefore, to our knowledge, these are the first reports to clarify the function of miR-200a-3p in the development of chicken skeletal muscle. 

MiRNAs regulate target genes by binding to their 3’-UTRs, thereby leading to their translational repression and/or degradation. We identified putative target genes of miR-200a-3p using three different software tools. Among the 45 most likely targets, we focused on *TGF-β2* because it is a known multifunctional cellular activity regulator [30]. Then, we confirmed that *TGF-β2* was a target gene of miR-200a-3p by a dual luciferase reporter system, qRT-PCR, and Western blot. A new study found that miR-200a-3p is a direct transcriptional repressor of *ZAK*, *MAP2K4*, and *TGF-β2* that are involved in the mitogen-activated protein kinase (MAPK) pathway to modulate an immune response in chicken afflicted with necrotic enteritis [31]. TGF-β2 belongs to the transforming growth factor family that has three members, TGF-β1, TGF-β2, and TGF-β3. These TGF-β isoforms have similar functional properties and regulate various cellular processes, including cell differentiation, apoptosis, and extracellular matrix synthesis [32]. Previous studies have shown that TGF-β2 inhibited differentiation of adult human skeletal myoblasts [33], and its expression was increased in skeletal muscles disorders [34]. The temporal and spatial expression of *TGF-β2* mRNA in somites and embryonic biceps femoris muscle suggested that TGF-β2 might be a regulatory factor in the somitogenesis and myogenesis of chicken embryos [35,36]. However, the exact function of TGF-β2 in chicken skeletal muscle development is still unclear. Our results suggested that TGF-β2 was a negative regulatory factor for chicken SMSCs because of its inhibitory effect on the differentiation and proliferation, and stimulation in apoptosis of SMSCs.

The TGF-β2/SMAD signaling pathway is known to play an important role in skeletal muscle development [37]. Canonical TGF-β2/SMAD signaling is dependent on the phosphorylation of SMAD2 and SMAD3 proteins, which then form a complex with SMAD4 to regulate the transcriptional activity of target genes [38]. Previous studies have reported that dysregulated expression of miRNAs affects the TGF-β2/SMAD signaling pathway to regulate biological processes, mainly those associated with tumorigenesis. For example: Howe et al. suggested that miR-30b regulated endothelial cell capillary morphogenesis by regulating *TGF-β2* expression [39]; Niu et al. found that miR-153 inhibited osteosarcoma cell proliferation and invasion by targeting *TGF-β2* [40]; Li et al. reported that miR-30a reversed TGF-β2-induced migration in posterior capsular opacification by targeting *SMAD2* [41]. However, to our knowledge, there is no related study on the involvement of miRNAs in skeletal muscle development by regulating TGF-β2/SMAD signaling. In this study, we showed that exogenous TGF-β2 induced the expression of p-SMAD2/3, but the miR-200a-3p mimic reduced this effect. In addition, the specific TGF-β2 inhibitor (ITD-1) effectively prevented the exogenous miR-200a-3p-modulated cell differentiation and apoptosis in SMSCs. These results demonstrated that the regulatory effect of miR-200a-3p on SMSCs was via the TGF-β/SMAD signaling pathway.

## 4. Materials and Methods 

### 4.1. Animals and Ethics Standards

Animal care and experiment procedures were approved by the Animal Welfare Committee of the Faculty of Agriculture at Sichuan Agriculture University at January 7, 2018, and the assurance number is 2018-177. Relevant guidelines and regulations were followed while performing all the methods.

### 4.2. Sample Collection and Total RNA Extraction

The fertilized eggs of Ross 308 and White Longhorn were incubated in the same conditions. After sex detection by PCR, only male chicks were selected for samples collection. The breast muscles were from 24 embryonic chicks including three individuals as biological replicates for broilers and layers at embryonic day 10 (E10), E13, E16, E19, respectively. Total RNA was extracted from breast muscles using a TRIzol reagent (Invitrogen, Carlsbad, CA, USA) according to the manufacturer’s instructions. The integrity and concentration of all RNA samples were analyzed by the RNA 6000 nanometer chip of RNA Bioanalyzer 2100 (Agilent Technologies, Santa Clara, CA, USA). All RNA samples were stored at −80 °C. 

### 4.3. Library Preparation for sRNA Sequencing

A total amount of 2.5 ng RNA per sample was used as input material for the RNA sample preparations. Sequencing libraries were generated using a NEBNext^®^ Ultra™ small RNA Sample Library Prep Kit for Illumina^®^ (New England Biolabs, Beverly, MA, USA) following the manufacturer’s recommendations and index codes were added to attribute sequences of each sample. Briefly, first of all, the 3′ SR adaptor and reverse transcription synthetic first chain were ligated, then PCR amplification and the size were selected. PAGE gel was used for electrophoresis fragment screening purposes, rubber cutting recycling as the pieces get small RNA libraries. At last, PCR products were purified by the AMPure XP system (Beckman Coulter, Brea, CA, USA) and the library quality was assessed on the Agilent Bioanalyzer 2100 system (Agilent Technologies).

### 4.4. Comparative Analysis

In order to filter out the filter ribosomal RNA (rRNA), transfer RNA (tRNA), small nuclear RNA (snRNA), small nucleolar RNA (snoRNA), and other ncRNA and Repbase sequences to obtain unannotated reads, the filtered clean reads were applied to the Silva database, GtRNAdb database, Rfamdatabase and Repbase database for sequence alignment by the software of Bowtie [42]. The remaining reads were used to detect known miRNAs and novel miRNAs predicted by comparing with known miRNAs from miRbase (www.mirbase.org). Randfold tools soft was used for novel miRNA secondary structure prediction. The expression levels of miRNAs in each sample were statistically analyzed and the expression levels were normalized using the TPM algorithm [43]. The TPM normalization formula was as follows:

(1) $$TPM=\frac{R\mathrm{eadcount}\times 1,000,000}{M\mathrm{apped}\;R\mathrm{eads}}$$

### 4.5. Target Gene Functional Annotation and Differential Expression Analysis

The gene function was annotated based on the following databases: Nr (NCBI nonredundant protein sequences); Nt (NCBI nonredundant nucleotide sequences); Pfam (Protein family); KOG/COG (Clusters of Orthologous Groups of proteins); Swiss-Prot (A manually annotated and reviewed protein sequence database); KO (KEGG ortholog database); GO (gene ontology).

Differential expression analysis of two conditions/groups was performed using the DESeq R package (1.10.1). DESeq provides statistical routines for determining differential expression in digital miRNA expression data using a model based on the negative binomial distribution. The resulting *p*-values were adjusted using the Benjamini and Hochberg’s approach for controlling the false discovery rate. MiRNA with an adjusted *p* < 0.05 found by DESeq were assigned as differentially expressed. The GO enrichment analysis of differentially expressed genes (DEGs) was implemented by the GOseq R packages based Wallenius noncentral hyper-geometric distribution. KEGG, a database resource for understanding high-level functions and utilities of the biological system, such as the cell, the organism, and the ecosystem from molecular-level information, especially large-scale molecular datasets generated by genome sequencing and other high-throughput experimental technologies (http://www.genome.jp/kegg/). 

### 4.6. Cell Culture and Transfection

The breast muscle of four-day-old ROSS 308 chicks was collected for primary isolation and culture of SMSCs. Muscles were first cut and then treated with 0.1% collagenase I (Sigma, St. Louis, MO, USA) followed by 0.25% trypsin (Hyclone, UT, USA) to release cells. The cell suspension was then filtered and subjected to Percoll density centrifugation to isolate myocytes. Cells were plated in 25 cm^3^ cell culture bottles with DMEM/F12 (Invitrogen, Carlsbad, CA, USA), 15% FBS (Gibco, Grand Island, NY, USA), 1% penicillin-streptomycin (Solarbio, Beijing, China), and 3% chicken embryo extraction. Cells were allowed to proliferate in a growth medium for 2–4 day and the medium was refreshed every 24 h. To induce differentiation, satellite cells are cultured in a differentiation medium consisting of DMEM/F12, 2% horse serum (Hyclone), and 1% penicillin-streptomycin (Solarbio) when cells grow to 80% confluence in a growth medium, and the medium was refreshed every 24 h. The chicken fibroblast cell line DF-1 was used for the double luciferase reporter assay, which were cultured in a 10% fetal bovine serum, then (Gibco) was added to Dulbecco’s Modified Eagle Medium (DMEM; Sigma) in an environment humidified at 37 °C and 5% CO_2_, and the medium was refreshed every 24 h.

MiR-200a-3p mimics, inhibitors, si-TGF-β2, or pcDNA3.1-TGF-β2 were transfected into satellite cells using Lipofectamine^®^ 3000 (Invitrogen) according to the manufacturer’s instructions and repeated three times per group. When the cell fusion rate reached about 80–90%, transfected cells were cultured in DM to study cell differentiation; 50–60% of transfected cells were cultured in GM to study cell proliferation, differentiation, and apoptosis. Diluted oligonucleotides or plasmids, Lipofectamine^®^ 3000, and diluted DNA using an Opti-MEM^®^ (Gibco) media were transfected into the cells. Samples were collected after 24 h (proliferation/apoptosis) or 48 h (differentiation) for further analysis.

### 4.7. Construction of Plasmids and RNA Oligonucleotides

MiR-200a-3p inhibitor, negative inhibitor, miR-200a-3p mimic, and negative mimic were purchased from RiboBio (RiboBio, Guangzhou, Guangdong, China). The three siRNAs and siRNA NC of chicken TGF-β2 were purchased from GenePharma (GenePharma, Shanghai, China). The detailed sequences are shown in Table S6. After restriction enzyme digestion, the coding sequence fragment of TGF-β2 was ligated to the pcDNA3.1 (+) vector using a T4 DNA ligase, and the successfully constructed vector was named pcDNA3.1-TGF-β2.

### 4.8. Extraction of RNA, Synthesis of cDNA, and Real-Time Quantitative PCR

Total RNA was extracted using a TRIzol reagent according to the manufacturer’s instructions. Thermo Scientific ™ NanoDrop Lite (Thermo, Waltham, MA, USA) was used to measure the integrity and concentration of RNA in the samples, and the subsequent experiments were performed after passing. Total RNA was stored at −80 ° C. Prime Script RT Master Mix Perfect Real-Time (Takara, Dalian, China) was used for mRNA cDNA synthesis and the miRNA reverse transcription reaction was performed using a one-step miRNA cDNA synthesis kit (HaiGene, Haerbin, Heilongjiang China).

The real-time PCR primer sequences were shown in Table S7 and designed by the Primer Premier 6 software (PREMIER Biosoft, San Francisco, CA, USA). The CFX96-Touch^TM^ real-time PCR detection system (Bio-Rad, Hercules, CA, USA) was used to detect the mRNA abundance. The reaction volume of real-time PCR includes 1 μL cDNA, 0.5 μL reverse and forward primers (per gene), 3 μL double distilled water, and 5 μLTB Green™ Premix Ex Taq™ II (Takara). All reactions were repeated three times. Relative gene expression was determined by the 2^−ΔΔCt^ method [44].

### 4.9. Immunofluorescence and Western Blot

Cells were fixed using 4% paraformaldehyde (Solarbio) according to the manufacturer’s instructions. The fixed cells were infiltrated with 0.5% Triton X-100 (Beyotime, Shanghai, China) for 20 min after washing with PBS (Hyclone) and blocked with goat serum (Solarbio) for 30 min. Next, the primary antibody was added and incubated overnight at 4 °C. The cells were washed with PBST (0.05% Tween 20; Chron Chemical, Chengdu, Sichuan, China) + PBS (Hyclone, South Logan, UT, USA) and a diluted secondary antibody was added and incubated at 37 °C for 1 h. Next, the nucleus was stained with DAPI (4′,6-diamidino-2-phenylindole; Solarbio) for 5 min in the dark. Images were captured by a fluorescence microscope (Olympus, Melville, NY, USA).

Detailed experimental methods for the Western blot analysis are described in depth by Shen et al. (2019) [45]. The antibodies used for the experiments were as follows: Anti-MyHC (Santa Cruz Biotechnology, CA, USA; 1:200 dilution), anti-MyoD (Santa Cruz Biotechnology; 1:500 dilution), anti-caspase-3 (Abcam, London, UK; 1:1,000 dilution),anti-caspase-9 (Abcam, London, UK; 1:1000 dilution), anti-TGF-β2 (ABclonal, Wuhan, Hubei, China; 1:1000 dilution), anti-β-actin (Santa Cruz Biotechnology; 1:1000 dilution), anti-SMAD2/3 and anti-p-SMAD2/3 (ABclonal, Wuhan, Hubei, China; 1:1000 dilution). β-actin was used as the loading control.

### 4.10. Prediction of Target Gene

Target genes of miR-200a-3p were predicted using three online tools, including miRDB (http://mirdb.org/), TargetScan (http://www.targetscan.org/vert_71/), and Diana (http://www.microrna.gr/microT-CDS).

### 4.11. Luciferase Reporter Assay

Fragments of miR-200a-3p, including the binding site of *TGF-β2*, were amplified and inserted into pEZX-FR02 vectors (GeneCopoeia, Amaranth Drive Germantown, Maryland, USA) at the 3′ end of the Firefly Luciferase gene using restriction enzymes BsiWI and XhoI (TaKaRa) and T4 DNA ligase (pEZX-*TGF-β2*-WT). Mutant pEZX-*TGF-β2*-MT was generated by mutating complementary to the seed region of miR-200a-3p using mutagenic primers. All constructs were verified by sequence analysis.

### 4.12. CCK-8 and EDU

Cell proliferation was measured using the Cell Count Kit-8 (CCK-8; Meilunbio, Shanghai, China) according to the manufacturer’s instructions and cell suspensions (100 μL/well) were seeded in 96-well plates. Next, the plate was placed in the incubator for a period of time (37 °C, 5% CO_2_), 10 μL of the CCK-8 solution was added to each well, and the plate was incubated for 1–4 h. Each experiment was repeated six times for each group. Absorbance was measured at 450 nm using a Thermo Scientific ™ Varioskan LUX. Four time points were taken for measurement, which were 12, 24, 48, and 72 h after transfection.

After transfection of cells, EDU experiments were performed using the C10310 EdU Apollo in vitro imaging kit (RiboBio) according to the manufacturer’s instructions. Cells were first incubated with an EdU medium and then washed with PBS. Next, the cells were then fixed with 4% paraformaldehyde and stained using a kit. Three areas were randomly selected using a fluorescence microscope to assess the number of stained cells.

### 4.13. Flow Cytometry Analysis of Apoptosis

The cells were centrifuged at 250 g for 5 min, the supernatant was discarded, and a 100 ul binding buffer was added to resuspend the cells. Annexin v-fitc (Invitrogen) with 5 μL was added for staining with a fluorescent dye for 10 min, avoiding light at room temperature. Subsequently, 10 μL of PI (BD Pharmingen, Santiago, CA, USA) was added with a fluorescent dye for 5 min, avoiding light at room temperature, and then 400 μL of a combined buffer suspension was added immediately on the machine for detection. The CytoFLEX flow cytometer (Beckman Coulter, Brea, CA, USA) was used for detection and Kaluza 2.1 software was used for data analysis.

### 4.14. Statistical Analysis

Statistical analyses were performed using the SPSS 19.0 Statistical software (SPSS, Inc., Chicago, IL, USA). Each experiment was repeated three times. The one-way analysis of variance or unpaired t-test was used to test statistical significance between groups. Data are presented as the least squares means ± standard error of the mean (SEM). Differences were considered significant at the *p* < 0.05 level. All the data are available in the SRA database with NCBI accession number PRJNA516545.

## 5. Conclusions

**Figure 11:**
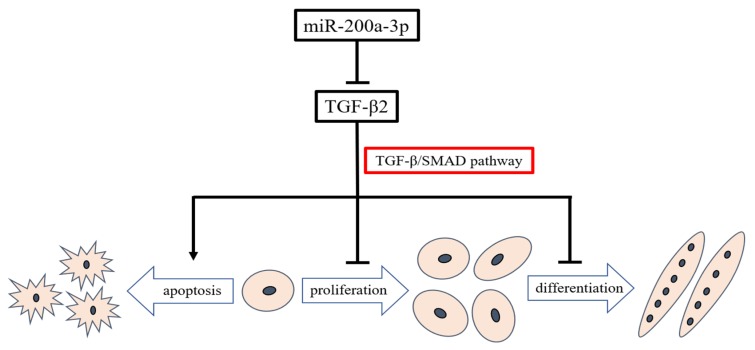
Model illustrating the miR-200a-3p-mediated regulatory pathway in chicken skeletal muscle satellite cells.

This study provides an overview of miRNAs expression in embryonic chicken skeletal muscle. We found that miRNAs were abundant and differentially expressed between layers and broilers during chicken embryo development. We confirmed that miR-200a-3p promoted SMSCs development by targeting *TGF-β2* and regulating the TGF-β/SMAD signaling pathway (Figure 11). We constructed a model that illustrates the miR-200a-3p regulatory role in chicken skeletal muscle development. This model may help better understand the role of miR-200a-3p in myogenesis and could pave the way for new therapeutic approaches in regenerative medicine.

## Figures and Tables

**Table 1:** Top 10 most abundant known miRNAs in chicken skeletal muscle.

miRNA Name		E10	E13	E16	E19	Mean TPM
gga-miR-148a-3p	broiler	327293	323059	364718	322018	334272
	layer	353929	339140	391420	343636	357031
gga-miR-1a-3p	broiler	26616	79775	126939	191575	106227
	layer	20129	64899	117576	195302	99476
gga-miR-206	broiler	27301	61997	48354	27326	41245
	layer	26028	57154	45154	32489	40206
gga-miR-199-3p	broiler	41384	28932	31157	28151	32406
	layer	46854	31789	24972	29883	33375
gga-miR-99a-5p	broiler	41622	37123	28133	20793	31918
	layer	46148	36928	27424	23673	33543
gga-miR-26a-5p	broiler	29754	24827	28814	38364	30440
	layer	29251	25395	24006	32410	27766
gga-let-7i	broiler	30227	33640	25192	24596	28413
	layer	25134	34570	28353	23014	27768
gga-miR-21-5p	broiler	43718	28132	17625	19367	27211
	layer	34517	25338	17125	11944	22231
gga-miR-100-5p	broiler	41356	24980	18641	13781	24690
	layer	32922	22507	15828	15976	21808
gga-let-7f-5p	broiler	17858	24193	21957	24043	22012
	layer	14383	22991	21583	21334	20073

**Table 2:** Top 10 most highly differentially expressed miRNAs (DEMs) in chicken skeletal muscle.

miRNA Name		E10	E13	E16	E19
gga-miR-9-5p	layer	2533.501309^a^	460.2524814	479.6634151	307.1783952
	broiler	725.8412491^b^	601.6528318	554.8960751	421.2946555
gga-miR-200a-3p	layer	587.7834844	108.5109794^a^	39.07409964^a^	65.79358588
	broiler	388.8403976	397.7650247^b^	130.4198329^b^	119.8415039
gga-miR-187-3p	layer	177.0353363	279.7273104^a^	167.3982378^a^	50.00691963^a^
	broiler	98.41777916	45.56077617^b^	10.18975533^b^	7.038522079^b^
gga-miR-215-5p	layer	64.26356133	69.62688177	53.95331555^a^	35.62181554
	broiler	110.8988222	97.79864923	146.497047^b^	43.44877201
gga-miR-383-5p	layer	72.01323789	44.89056409^a^	48.3486871	25.54511817
	broiler	104.8076691	113.1937947^b^	78.04010784	54.44535269
gga-miR-3525	layer	95.6159864^a^	83.03892403	49.4126213	30.16373365
	broiler	43.59036593^b^	58.00610376	27.19807371	19.32445908
gga-miR-122-5p	layer	46.01366404	37.70219446	29.61562344	152.9681583^a^
	broiler	61.89245507	26.78922018	23.62469575	26.27846184^b^
gga-miR-217-5p	layer	36.87902642	33.87950711^a^	15.41982366^a^	18.98201401^a^
	broiler	36.65022205	69.46941821^b^	39.87922917^b^	39.67657653^b^
gga-miR-490-3p	layer	68.14927667	55.23690091	12.01854442	2.781629621^a^
	broiler	63.45003703	34.82351892	11.40091401	5.668242481^b^
gga-miR-429-3p	layer	70.3618033	45.63204794^a^	11.19323588	6.365474227
	broiler	44.40631114	14.12317871^b^	3.330957988	11.92037763

^a,b^ means miRNAs are differentially expressed in broiler and layer chicken during this embryonic period.

## Supplementary Materials

The following are available online at https://www.mdpi.com/1422-0067/21/9/3274/s1, Figure S1. GO and KEGG analysis of differentially expressed miRNA target genes; Table S7. Primers used for quantitative real-time PCR.
