# Supplementary material for: MicroRNA Profiling Reveals an Abundant miR-200a-3p Promotes Skeletal Muscle Satellite Cell Development by Targeting TGF-β2 and Regulating the TGF-β2/SMAD Signaling Pathway
Source: Int J Mol Sci. 2020 May 5;21(9):3274. doi: 10.3390/ijms21093274 (PMC7247338; doi:10.3390/ijms21093274)
Supplement: Supplementary file 1 [file ijms-21-03274-s001.zip › Supplementary Figures.docx]

**
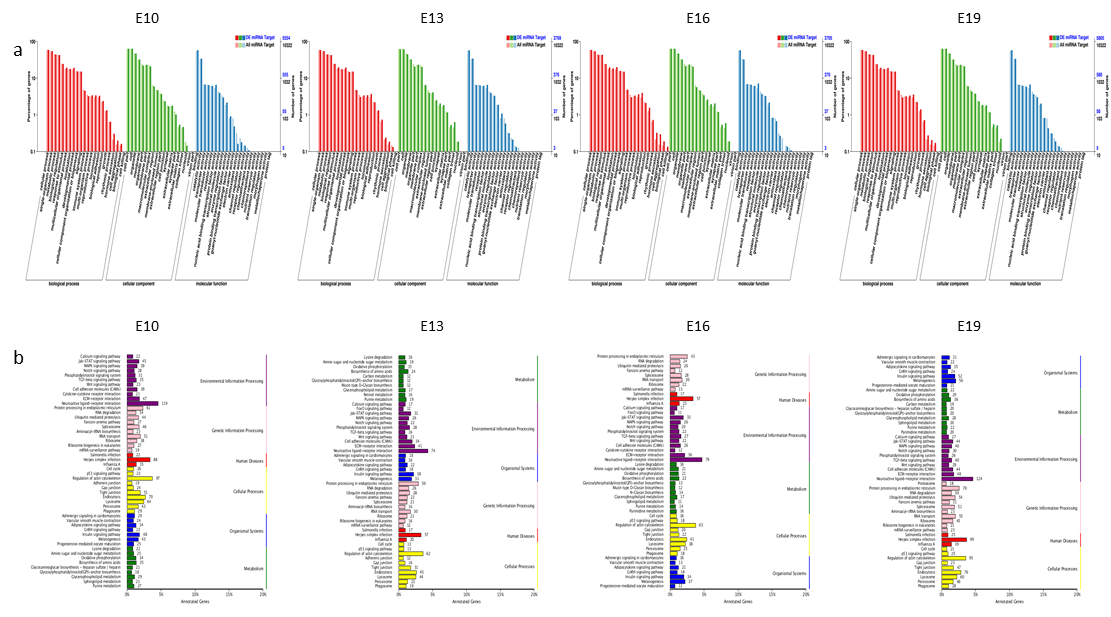
Figure S1. GO and KEGG analysis of differentially expressed miRNA target genes.** (a) GO analysis (biological process, cellular component and molecular function) of differentially expressed miRNA target genes. (b) KEGG analysis of differentially expressed miRNA target genes.
